# Experimental Research on Mechanical Properties and Compression Constitutive Relationship of PVA Fiber-Reinforced Coral Concrete

**DOI:** 10.3390/ma15051762

**Published:** 2022-02-26

**Authors:** Lan Rao, Ling Wang, Yun Zheng

**Affiliations:** 1 School of Civil Engineering, Xi’an University of Architecture & Technology, Xi’an 710055, China; wangling_mcc@163.com; 2 School of Civil and Architectural Engineering, Kaifeng University, Kaifeng 475004, China; 3 Central Research Institute of Building and Construction Co., Ltd., MCC, Beijing 100088, China; nrcsc@139.com

**Keywords:** coral concrete, PVA fiber, mechanical property, stress–strain curve, constitutive relationship

## Abstract

In this paper, the mechanical properties of coral concrete with different strength and different polyvinyl alcohol (PVA) fiber content under compression were experimentally investigated. The results show that adding an appropriate amount of PVA fiber could obtain satisfactory mechanical properties of coral concrete. The stress–strain constitutive relationship of plain and PVA fiber-reinforced coral concrete was investigated by prism uniaxial compression test. The results shown that the incorporation of PVA fiber had a significant effect on limiting the development of concrete internal cracks, and effectively improved the mechanical properties of coral concrete after cracking, especially the toughness. Different constitutive models from previous research were used to describe the axial compressive stress–strain relationship of plain and PVA fiber-reinforced coral concrete, and a piecewise function model was finally selected which is most consistent with the experimental curve and its characteristic points. In addition, determination of critical parameters for the selected constitutive model was proposed, and experimental validations confirmed its accuracy.

## 1. Introduction

In recent years, many countries have increased their investment in ocean engineering in response to the increasing depletion of land resources. For example, the construction of artificial islands is receiving more attention in many coastal countries, and the sea sand-broken reef aggregate concrete material is also becoming a research focus in construction materials. For off-shore islands and reefs lacking traditional construction materials, the problem of cost increase is inevitable and unacceptable. Fortunately, the coral resources in many off-shore islands and reefs are rich. Therefore, inventing a new kind of concrete with the best use of coral resources becomes a potential approach in off-shore islands and reef engineering construction.

As early as the 1950s, the application and research of coral concrete had been started in the United States. Dempsey [1] and Narver [2] described the configuration requirements of coral concrete, and evaluated the coral concrete buildings. On some islands, such as Midway, Wake and Bikini, coral concrete was used to build airports and roads, and some of them are still in service [3]. In 1974, Howdyshell [4] investigated the coral concrete buildings, and confirmed that it was feasible to use coral aggregate instead of traditional coarse aggregate to prepare concrete. In 2015, Yuan [5] prepared high performance coral concrete with strength up to 60 MPa using fly ash and slag instead of part of P∙Ⅱ52.5 ordinary Portland cement in the mix proportion of coral concrete. So far, many researches have been conducted on the mix proportion, strength and durability of coral concrete [6,7]. However, there are few researches completed in terms of its constitutive relationship, especially the constitutive relationship of fiber-reinforced coral concrete. As the constitutive relationship of concrete is one of the most important factors that determine the servicing capacity of concrete engineering structures, the existing research results of coral concrete are still not enough for detailed engineering application or performance-based engineering design, and further research is needed.

In 2000, Sun [8] studied the mechanical properties of coral aggregate and pointed out that coral particles had great compressibility and were prone to being broken in the process of compression and shear because of its high porosity, which might be the major factor affecting the ductility of coral concrete, and lead to its mechanical performance significantly different from ordinary aggregate concrete, light aggregate concrete and recycled aggregate concrete [9,10,11,12,13]. It deserved more research to improve the ductility of coral concrete so as to better meet the ductility demand of engineering structures. In recent years, many scholars have carried out researches on fiber reinforced concrete, such as Mukhopadhyay [14], Pereira [15], Nia [16], and Yoo [17], which shown that fibers had significant effects on the crack resistance, toughness, impact resistance and other performances of the concrete. From 2014 to 2016, Wang [18,19,20], studied the basic mechanical indices of coral concrete mixed with carbon fiber, sisal fiber and polypropylene fiber, respectively. The results show that the fiber significantly improved the mechanical properties of coral concrete.

In general, due to the particularity of the marine environment, the durability of concrete structures with different kinds of fibers is obviously different. Coral concrete, as a special building material in the marine environment, not only requires the fiber to play a role of toughening and strengthening, but also to present the properties of weather resistance and corrosion resistance etc. Therefore, compared with other fibers, polyvinyl alcohol (PVA) fiber with low price and excellent performance might be a better choice.

PVA fiber is a kind of synthetic fiber with many advantages, such as high strength, high elasticity, good wear resistance, strong acid-base resistance, and good corrosion resistance [21]. Due to its strong affinity with cement base material and non-toxic or pollution characteristics, it has become one of the new generations of high-tech green building materials. Existing research results show that the addition of PVA fiber could effectively improve the strength, ductility [22,23], crack resistance [24] and durability [21] of concrete. However, there are few investigations on PVA fiber-reinforced coral aggregate concrete, which will be investigated in this study to supplement.

In this study, the experiments on cube compressive strength, prism axial compressive strength of plain and PVA fiber-reinforced coral concrete were carried out. Based on the experiment results, the influences of PVA fiber on the compression strength, elastic modulus and Poisson’s ratio of coral concrete were studied. Furthermore, the compression stress–strain curve characteristics of plain and PVA fiber-reinforced coral concrete were comparatively studied, and the constitutive model for uniaxial compression stress–strain curve of plain and PVA fiber-reinforced coral concrete was also proposed. The results might be referable for engineering application or performance-based engineering design.

## 2. Materials and Methods

### 2.1. Materials and Properties

**Figure 1:**
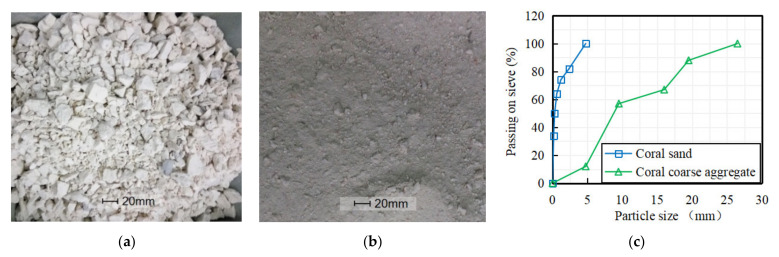
Coral aggregate from a South China Sea island. (**a**) Coarse aggregate, (**b**) Fine aggregate, (**c**) Size distribution of coral aggregate.

The coral from a Southern China Sea island was broken into irregular particles with a maximum diameter of 26.5 mm. After sieving, aggregate with continuous particle size of 4.75 to 26.5 mm was used as coarse aggregate (Figure 1a), and particles with continuous particle size less than 4.75 mm was used as fine aggregate (Figure 1b), which had a fineness modulus of 1.7. According to ASTM C33/C33M-2018 [25] and GB/T 17431.2-2010 [26] the particle size distribution and the physical properties of coral aggregate were measured as shown in Figure 1c and Table 1.

Ordinary Portland Cement P. O 42.5 (OPC), slag (SG) and silica fume (SF) were used in the tested mixtures, their chemical compositions are shown in Table 2. In this test, the coral concrete was also mixed with PVA fiber, the details of PVA fiber’s physical properties are shown in Table 3. According to the literature [27], artificial seawater was prepared according to the composition of the seawater in the Southern China Sea, and its chemical composition is given in Table 4. Polycarboxylate superplasticizer (SP) with solid content of 40% was used in the test.

### 2.2. Mix Proportion

According to Chinese technical specification for lightweight aggregate concrete (JGJ51-2002) [28] and Chinese standard for test methods for fiber reinforced concrete (CECS13: 2009) [29], different mix proportions were designed in this test. Through the equal quality substitution method, silica fume with substitution rate (by weight) of 5–7.5% and slag with substitution rate of 15–22.5% were used to replace part of cement to prepare coral concrete. Based on C40 specimen group, the mix proportions of coral concrete with different fiber content were designed, and compared with the reference group (C40), the fiber groups (FC1–FC4) were only different in fiber content, the fiber dosages were 0.8125 kg/m^3^, 1.625 kg/m^3^, 3.25 kg/m^3^ and 6.5 kg/m^3^, respectively, as shown in Table 5.

### 2.3. Specimen Production

Due to the water-absorption and water-release characteristics of coral aggregates [8,30], the problem of high drying shrinkage of coral concrete is prominent [10], however, investigations suggested that pre-wetting aggregate and adding shrinkage reducing agent could effectively inhibit the shrinkage deformation of coral concrete [31]. Hence, the coral aggregate was pre wetted during the preparation of coral concrete [32], and the preparation procedure of coral concrete was formulated as follows: (1) Added the coarse and fine gravel aggregate of coral coarse into the mixer and stirred for 1 min; (2) Added about 50% of the seawater which could be absorbed by the aggregate and stirred for 1–2 min; (3) Added the cementitious material and stirred for 1 min; (4) Added PVA fibers evenly to the mix while stirring, and continuously stirred for 1–2 min; (5) Added the polycarboxylate superplasticizer into the remaining water and mixed well, and then added it to the mixture in 2–3 times and mixed continuously for 1–2 min. The slump and dispersion of coral concrete were measured immediately after mixing. After the concrete was placed in molds of specific size, vibrated on a vibration table, the exposed surface concrete was covered with plastic film to prevent water dispersion, and then all specimens were demolded after curing under normal temperature (20 ± 3 °C) for 24 h. Finally, all the pieces were placed in the standard curing room for 28 days until being tested.

The test methods of mechanical properties of coral concrete were conducted according to Chinese standard for method of mechanical properties on ordinary concrete (GB/T 50081-2002) [33], and eight groups of specimens were prepared. Each group included nine specimens, of which three specimens with dimensions of 100 mm × 100 mm × 100 mm were used to test the cubic compressive strength, three specimens with dimensions of 100 mm × 100 mm × 300 mm were used to test the prism axial compression, and three specimens with dimensions of 100 mm × 100 mm × 300 mm were used for the uniaxial compressive stress–strain curve test.

### 2.4. Test Procedures

**Figure 2:**
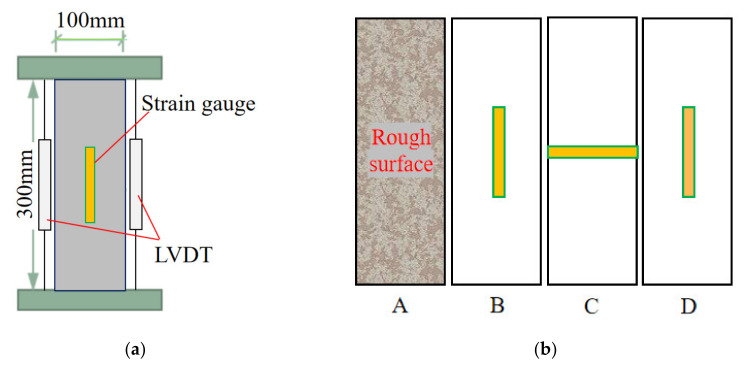
Test device and strain gauge arrangements. (**a**) Schematic diagram of test device, (**b**) Strain gauge diagram.

The electro-hydraulic servo universal testing machine (WAW-2000D, Jinan Zhonglu Chang testing machine Manufacturing Co., LTD, Jinan, China) was used for uniaxial compression test, with a maximum capacity of 2000 kN, as shown in Figure 2. During the compression strength test, the loading speed was kept in a uniform loading speed of 0.5 kN/s until the failure. During the loading of stress–strain curve test, the first step was to preload with 1 kN/s to 15% of the estimated ultimate load and then unload. This step was repeated three times. The second step was to check the strain collection values of two sides of the test specimens to ensure alignment. After three preloads, keep loading to 60% of the estimated ultimate load at the speed of 1 kN/s, and then continued to load until failure at the speed of 0.025 mm/min.

Three rubber-based strain gauges were pasted on each stress–strain curve test specimen, in which two longitudinal strain gauges were arranged at sides B and D, and one transverse strain gauge at side C, respectively, as shown in Figure 2b (as the specimen is molded, the surface side A is rough and uneven, so no strain gauge is set on side A), connected with the computer for synchronous strain acquisition before peak load, in addition, two linear voltage-displacement transducers (LVDT) were arranged in the both sides of the specimen to record the axial deformation. During the loading process, the load values of the test specimen were measured by the machine through the pressure sensors, then according to the measured data the computer automatically generated the curve of loading and unloading. The phenomenon was recorded during the test process. Due to the low strength and high brittleness of coral aggregate, the specimen generally cracked and then collapsed rapidly when it was loaded around the peak load, and therefore displacement gauge could just collect few data during the post peak stage.

## 3. Results and Discussion

### 3.1. Basic Physical and Mechanical Properties

#### 3.1.1. Working Performance

**Figure 3:**
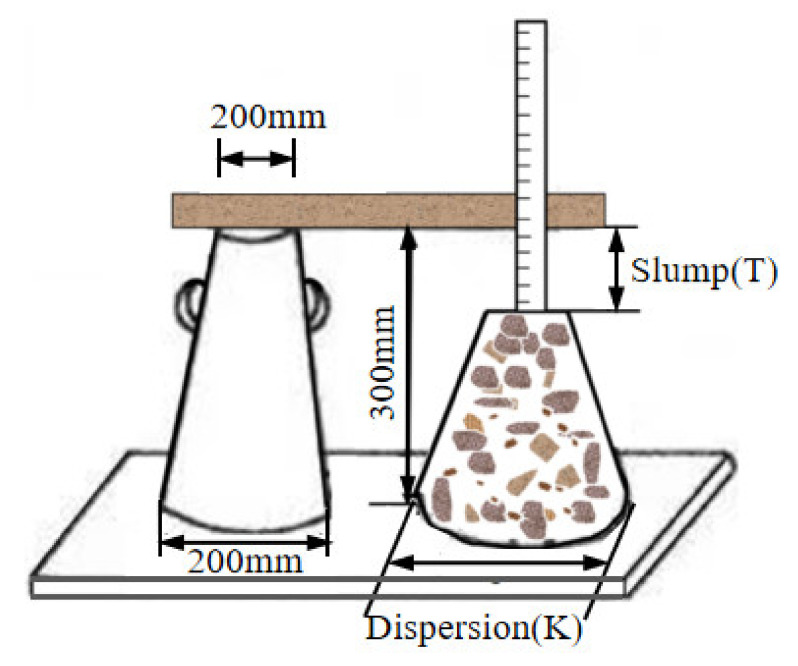
Slump and dispersion test method.

**Figure 4:**
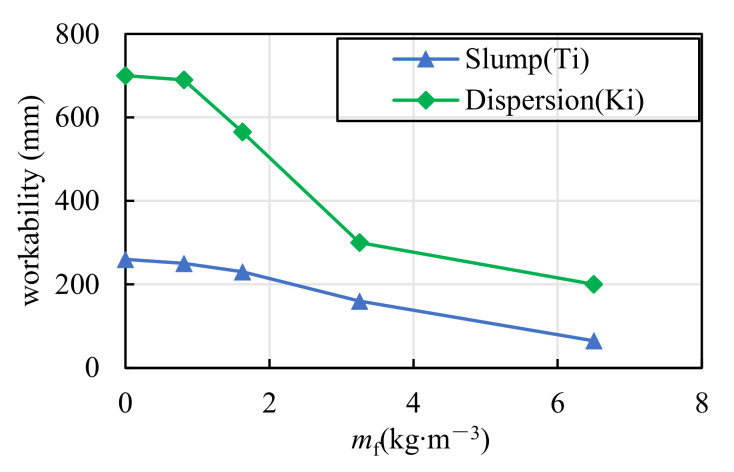
Effect of fiber content on concrete workability.

The tests of slump and dispersion were conducted according to Chinese standard GB/T 50080-2016 [34], as shown in Figure 3. The workability performance of coral concrete was classified according to Chinese standard GB50164-2011 [35], as shown in Table 6. Table 7 shows the original data measured in the test. In Figure 4, the curves show the relationship between concrete workability (slump $T_{i}$ or dispersion $K_{i}$) and fiber content ($m_{f}$).

From Table 7, the workability of all plain coral concrete groups in this paper was very good, yet it can be seen that the slump and dispersion of coral concrete show a rapid downward trend indicating that the viscosity of coral concrete significantly increased with the increase of PVA fiber content (as shown in Figure 4). Moreover, when the PVA fiber content increased to $V_{f}=6{.5\mathrm{kg}/m}^{3}$, the coral concrete’s fluidity was almost completely lost, and the slump value decreased to 65 mm, only 25% of the reference group (C40). This is mainly due to the following two reasons. On the one hand, PVA fiber has a large specific surface area and good cement compatibility; with the increase of fiber content, more and more cement slurry is used to wrap the fiber, resulting in an increased cohesion of coral concrete. On the other hand, the disordered fibers scattered in the concrete form a bridging effect, which hinders the flow of coral concrete more with the increase of fiber content. As the slump of concrete shall not be less than 70 mm during construction in high temperature environment [36], it is suggested that the PVA fiber dosage of coral concrete should not exceed 5 kg/m^3^ in marine environment construction. Besides, the dispersion of pumped concrete should not be less than 500 mm [35], and thus it is suggested that the PVA fiber dosage should not be more than 2.0 kg/m^3^ if using pumping construction.

#### 3.1.2. Test Phenomenon

During compression loading, almost all of the specimens shown obviously brittle failure. Most of the specimens began to appear obvious destructive cracks after bearing about 90% of the ultimate load. With the load approaching the peak load, the cracks spread rapidly and some of the concrete at the corners of the test specimens was torn into slag and dropped. Finally, the test specimen was destroyed immediately with a sound of cracking.

**Figure 5:**
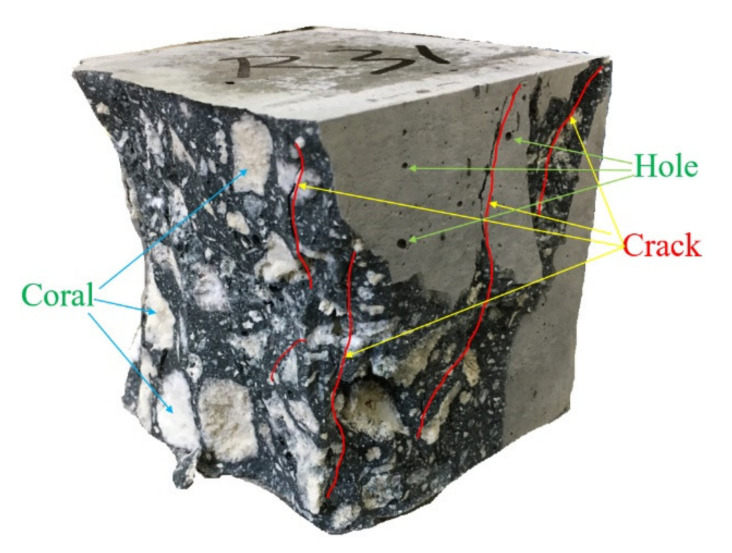
Typical failure modes of specimens in cube compression tests.

The typical failure of plain coral concrete specimen was mainly compression crushing, as shown in Figure 5, and some specimens were directly decomposed into several blocks by a few major cracks. Different from the crack propagation around the aggregate of ordinary concrete, all cracks in coral concrete specimens directly initiated and propagate through the coral aggregate, which indicated that the low strength and high porosity of coral aggregate leaded to the high brittleness of coral concrete. In addition, there are many visible holes of different sizes in the interior and surface of the coral concrete blocks with maximum diameter of about 2–3 mm. This may be due to the high porosity of coral aggregate, which exhausts the gas in the internal pores during water absorption.

**Figure 6:**
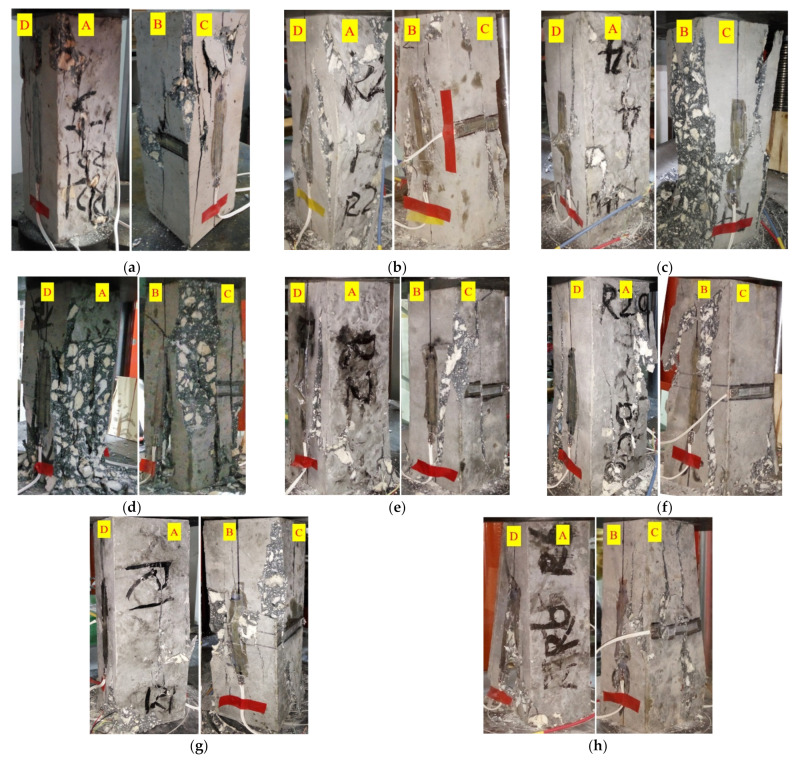
Failure modes of specimens in uniaxial compression stress–strain curve tests. (**a**) C25, *m*_f_ = 0 kg·m^−3^; (**b**) C30, *m*_f_ = 0 kg·m^−3^; (**c**) C35, *m*_f_ = 0 kg·m^−3^; (**d**) C40, *m*_f_ = 0 kg·m^−3^; (**e**) FC1, *m*_f_ = 0. 8125 kg·m^−3^; (**f**) FC2, mf = 1.625 kg·m^−3^; (**g**) FC3, *m*_f_ = 3.25 kg·m^−3^; (**h**) FC4, *m*_f_ = 6.5 kg·m^−3^.

However, the brittleness of coral concrete was obviously improved by adding PVA fiber. Compared with the plain coral concrete group (C40), the integrity of the specimen with PVA fibers (FC1 to FC4) was better due to the bridging and toughening effect of PVA fiber, and the local chipping and peeling was also significantly reduced, some specimens still remained integrity after failure, as shown in Figure 6.

#### 3.1.3. Mechanical Properties

The mechanical properties of experimental specimens are summarized in Table 8, and the measurement results are taken as the average of the three samples in each group. With comparison of specimens C40 and FC1 to FC4, it can be seen that adding an appropriate amount of PVA fiber can improve the compressive strength, peak strain, Poisson’s ratio, and residual stress after crushing failure also increased significantly. Yet, the effect of PVA fiber content on the elastic modulus of coral concrete is less than 3.5%.

In this paper, the slope of the linear segment between two points ($\left(\varepsilon_{1},\sigma_{1}\right)$ and $\left(\varepsilon_{2},\sigma_{2}\right)$) of the ascent section of the longitudinal stress–strain curve was taken as the elastic modulus $E_{c}$ [37,38] of coral concrete, and this method is reasonable. The formula is as follows:

(1) $$E_{c}=(\sigma_{2}-\sigma_{1})/(\varepsilon_{2}-\varepsilon_{1})$$

where $\sigma_{1}=0.2\sigma_{\mathrm{cr}},\sigma_{2}=0.4\sigma_{\mathrm{cr}}$, $\varepsilon_{2}$ and $\varepsilon_{1}$ are the longitudinal strains corresponding to $\sigma_{2}$ and $\sigma_{1}$ on the stress–strain curve, respectively [38].

Poisson’s ratio is defined as the absolute value of the ratio of the transverse strain to the longitudinal strain of the specimen [39]. In this paper, the ratio of the transverse strain to the longitudinal strain of two points ($\left(\varepsilon_{1},\varepsilon_{t1},\sigma_{1}\right)$ and $\left(\varepsilon_{2},\varepsilon_{t2},\sigma_{2}\right)$) corresponding to $E_{c}$ is taken as Poisson’s ratio, and the formula is as follows:

(2) $$\mu =(\varepsilon_{t2}-\varepsilon_{t1})/(\varepsilon_{2}-\varepsilon_{1})$$

where $\varepsilon_{t1}(\varepsilon_{t2})$ and $\varepsilon_{1}(\varepsilon_{2})$ are the transverse strain and longitudinal strain corresponding to the stress of $0.2\sigma_{\mathrm{cr}}$ and $0.4\sigma_{\mathrm{cr}}$, respectively, on the ascend stage of the uniaxial compression stress–strain curve.

### 3.2. Discussions on Uniaxial Compression Stress–Strain Curves

#### 3.2.1. Test Results

According to Equations (3) and (4), the measured load and longitudinal displacement were converted into stress σ and strain ε.

(3) σ=P/A

(4) $$\varepsilon =\left\{\begin{array}{l} (\varepsilon_{L}+\varepsilon_{R})/2\\ \Delta L/L \end{array}\right.\begin{array}{l} \;\;\;\;\;\;\;\;\;\;\;\;(\sigma \leq \sigma_{\mathrm{cr}})\\ \;\;\;\;\;\;\;\;\;\;\;\;(\sigma >\sigma_{\mathrm{cr}}) \end{array}$$

where σ is stress of concrete specimen (MPa); P is axial pressure (N); A is concrete specimen compression area (mm^2^); ε is longitudinal strain of concrete specimen; L is the longitudinal displacement (mm); ΔL is longitudinal deformation (mm); $\varepsilon_{L},\varepsilon_{R}$ are longitudinal strains measured by the longitudinal strain gauges on the B side and D side of the specimen (see Figure 2b) before loading to the ultimate load.

In order to analyze the stress–strain curve and toughness index accurately, the author normalized the measured data as follows:

Firstly, the stress values and the strain values of the stress–strain curve were divided by the peak stress and peak strain, respectively, as follows:

(5) $$y=\sigma /\sigma_{cr}$$

(6) $$x=\varepsilon /\varepsilon_{cr}$$

where $\sigma_{\mathrm{cr}}$ is peak stress (MPa); $\varepsilon_{\mathrm{cr}}$ is peak strain.

Secondly, on the basis of the equal or close strain values ($x_{1i}\approx x_{2i}\approx x_{3i}$), calculating the weighted average of the stress values of three specimens from the same group (the weighting coefficient is 1/3), i.e., as follows:

(7) $$x_{i}^{}=\frac{x_{1i}+x_{2i}+x_{3i}}{3}$$

(8) $$y_{i}^{}=\frac{y_{1i}+y_{2i}+y_{3i}}{3}$$

where $x_{1i},x_{2i},x_{3i}$ are the relative strain values (dimensionless) of three samples in the same group respectively, $y_{1i},y_{2i},y_{3i}$ are the relative stress values (dimensionless) corresponding to $x_{1i},x_{2i},x_{3i}$, respectively.

**Figure 7:**
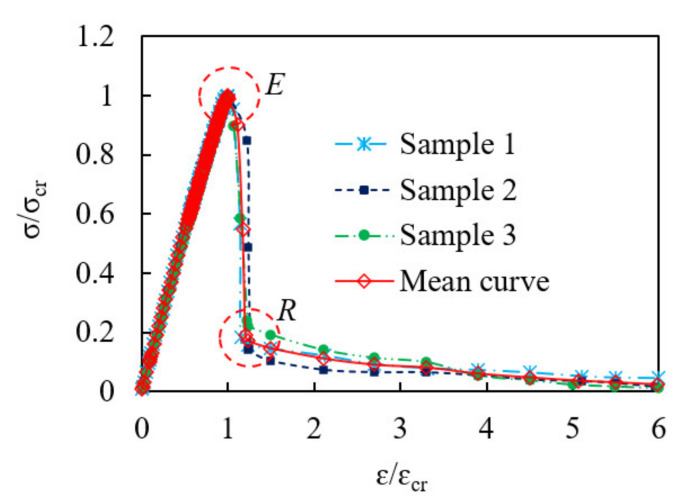
Standardization of stress–strain curve (CF4).

In order to get accurate and effective normalized stress–strain curve, it is necessary to pay attention to the continuous transition around the peak point (E) and residual point (R) to ensure the consistency with the original curve shapes, taking group 5 as an example, shown in Figure 7.

**Figure 8:**
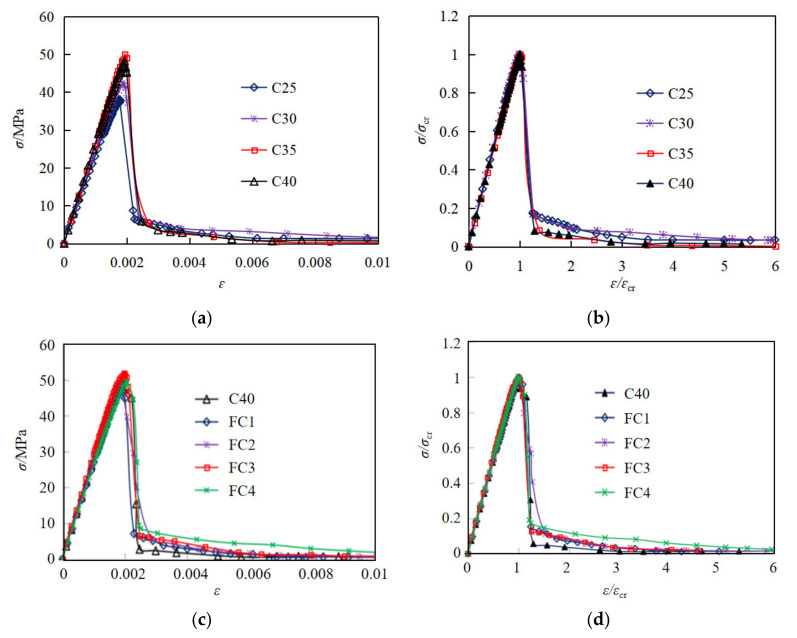
Comparative stress–strain relationship for coral concrete with different strengths and fiber content. (**a**) stress–strain relationship for coral concrete with different strengths, (**b**) normalized stress–strain relationship for coral concrete with different strengths (dimensionless), (**c**) stress–strain relationship for coral concrete with different fiber content, (**d**) normalized stress–strain relationship for coral concrete with different fiber content (dimensionless).

Finally, according to the above steps, the normalized curves of all test groups were obtained, and the stress and strain values (dimensionless) of the normalized stress–strain curves were multiplied by the average peak stress and average peak strain of the same group, respectively, so as to obtain the average stress–strain curves of each group, as shown in Figure 8.

As shown in Figure 8, before 90% of the peak stress, the slope is constant, indicating that the strain increases linearly with the stress. As the stress approaches 90% of the peak stress, some visible cracks begin to appear on the surface of concrete and then develops rapidly. Afterwards, with increasing the stress up to ultimate stress, the curves start to clearly deviate from the straight line. When the stress exceeds the ultimate stress, the stress decreases sharply and then the specimens are crushed rapidly. As a result, only a few data points in the declining stage of curves could be collected due to the rapid failure process.

With an increase of concrete strength, the slope of stress–strain relationship increases correspondingly, indicating the elastic modulus of coral concrete increases, and the peak strain also increases, as shown in Figure 8a. However, the residual stress generally decreases with increasing concrete strength, as shown in Figure 8a and Table 8. From the normalized stress–strain relationship in Figure 8b, it can be seen that the curves of coral concrete with different strengths almost coincide in the linear part before 90% of the peak stress, thus it can be concluded that the elastic modulus of coral concrete may have an approximate linear relationship with the strength.

As can be seen from Figure 8c and Table 8, compared with the control group (C40), when the content of PVA fiber was 0.8125 kg/m^3^,1.625 kg/m^3^,3.25 kg/m^3^, 6.5 kg/m^3^, the peak stress of coral concrete increased by −3.1%, 3.1%, 7.3%, 2.5%, the peak strain increased by −1.7%, −2.5%, 4.7%, 8.0%, the residual stress after failure increased by 27.1%, 20.3%, 25.4%, 66.1%, and the Poisson’s ratio increased by 6.7%, 23.8%, 25.8%, 3.3%, respectively, indicating that the addition of an appropriate amount of PVA fiber had a positive toughening effect. However, the initial elastic modulus was rarely influenced by fiber content before 50% of the peak stress. In addition, the shape and the feature point of the normalized stress–strain curves of the coral concrete with different fiber content had a minor difference.

#### 3.2.2. Toughness Index

**Figure 9:**
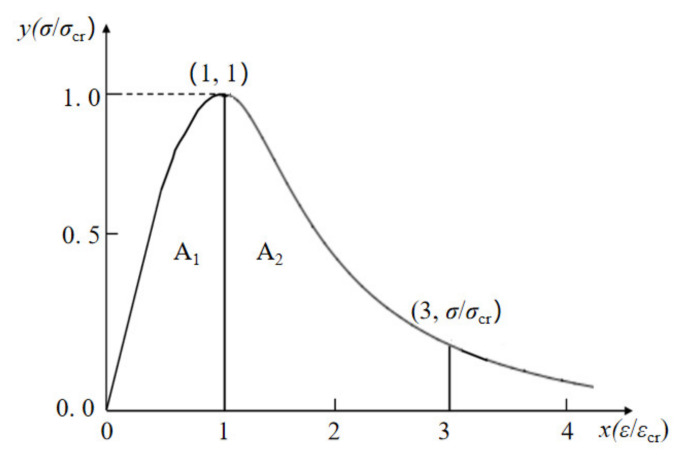
Feature points of toughness index.

Toughness is an important index to evaluate the deformation ability of materials or structures after reaching the ultimate load [40]. Referring to the evaluation method of concrete toughness index [41] and some suggestions from literature [42], the paper used the area parameters under the stress–strain curve of uniaxial compression (normalized) to describe the toughness, with the peak point $\left(\sigma_{cr},\varepsilon_{cr}\right)$ as the first feature point and the $3\varepsilon_{cr}$ point as the second characteristic point, as shown in Figure 9. The formulas are given as follows:

(9) $$\delta =\frac{A_{1}+A_{2}}{A_{1}}=\frac{\int_{0}^{3\varepsilon_{cr}}\sigma d\varepsilon }{\int_{0}^{\varepsilon_{cr}}\sigma d\varepsilon }=\frac{(\sigma_{cr}\times \varepsilon_{cr})\int_{0}^{3}yd\varepsilon }{(\sigma_{cr}\times \varepsilon_{cr})\int_{0}^{1}yd\varepsilon }=\frac{\int_{0}^{3}yd\varepsilon }{\int_{0}^{1}yd\varepsilon }$$

(10) $$\beta =\frac{A_{1}}{A_{2}}=\frac{\int_{0}^{\varepsilon_{cr}}\sigma d\varepsilon }{\int_{\varepsilon_{cr}}^{3\varepsilon_{cr}}\sigma d\varepsilon }=\frac{(\sigma_{cr}\times \varepsilon_{cr})\int_{0}^{1}yd\varepsilon }{(\sigma_{cr}\times \varepsilon_{cr})\int_{1}^{3}yd\varepsilon }=\frac{\int_{0}^{1}yd\varepsilon }{\int_{1}^{3}yd\varepsilon }=\frac{1}{\delta -1}$$

where *δ* is the toughness index of coral concrete; *β* is the brittleness index of coral concrete; $A_{1}$ is the area enclosed by the stress–strain curve in the strain $\left(0-\varepsilon_{cr}\right)$ interval, which represents the energy density absorbed before concrete failure; $A_{2}$ is the area enclosed by the stress–strain curve in the strain $\left(\varepsilon_{cr},3\varepsilon_{cr}\right)$ interval, which represents the energy density released during the concrete failure process.

**Figure 10:**
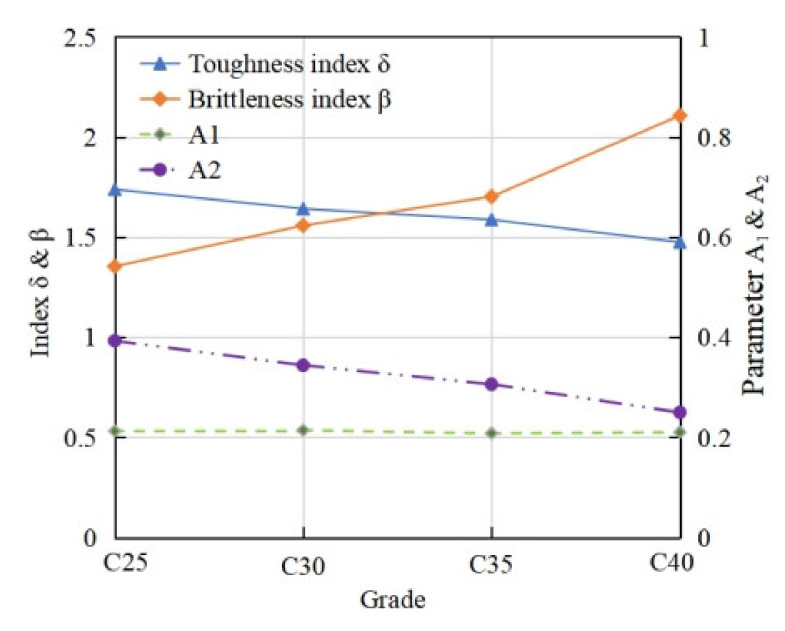
Toughness of coral concrete with different strengths.

As shown in Figure 10, with increase of concrete strength, the area parameter *A*_2_ decreases obviously while the area parameter *A*_1_ rarely changes. Thus, the corresponding toughness index *δ* decreases and brittleness index *β* increases.

**Figure 11:**
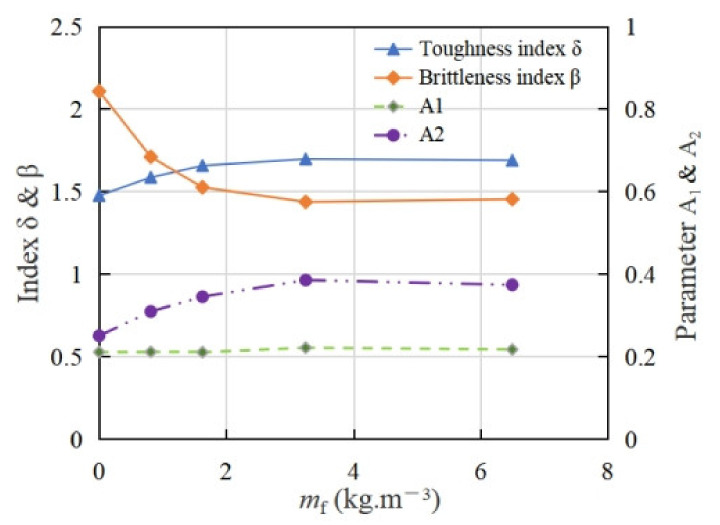
Toughness of coral concrete with different fiber contents.

As shown in Figure 11, the area parameter *A*_1_ of fiber reinforced coral concrete increased slightly, but the influence of different fiber content is not obvious among the different specimen fiber contents. However, the fiber has significant influence on area parameter *A*_2_. Compared with group C40 without fiber, when the fiber content is 0.8125 kg/m^3^, 1.625 kg/m^3^, 3.25 kg/m^3^ and 6.5 kg/m^3^, the parameter *A*_2_ increases by 23.6%, 37.9%, 54.0% and 49.4%, respectively, and the toughness index *δ* increased by 7.5%, 12.3%, 15.0% and 14.5%, respectively. Correspondingly, the brittleness index *β* declined by 18.9%, 27.6%, 31.8% and 31.1%, respectively, which shows that the ductility of the coral concrete is effectively improved by adding fiber.

#### 3.2.3. Numerical Modeling

The existing literatures show that there is no unified conclusion about the expression of the uniaxial compression stress–strain curve of coral concrete. Based on the existing concrete constitutive models and the test data, there is no unified constitutive models to describe the compression behavior of coral concrete. Considering the significant differences in the stress–strain curve between the ascending and declining stages of coral concrete, piecewise functions are used to describe the curve.

**Figure 12:**
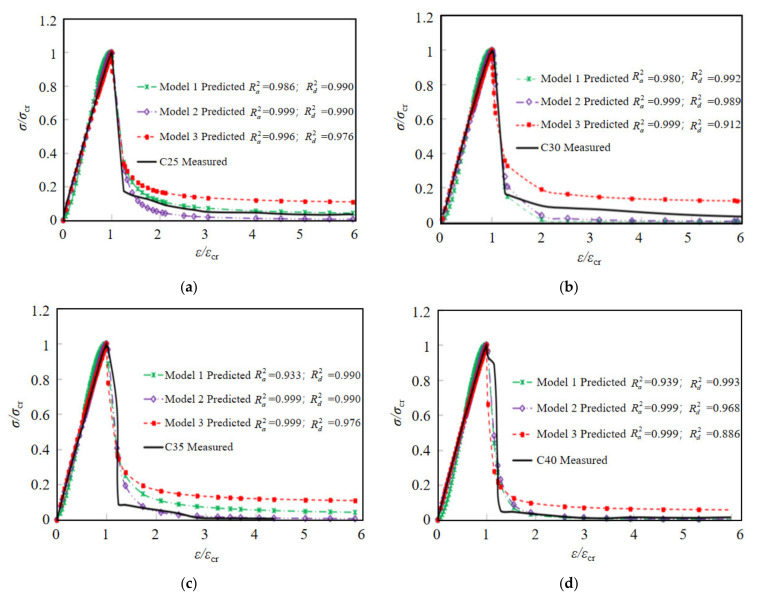

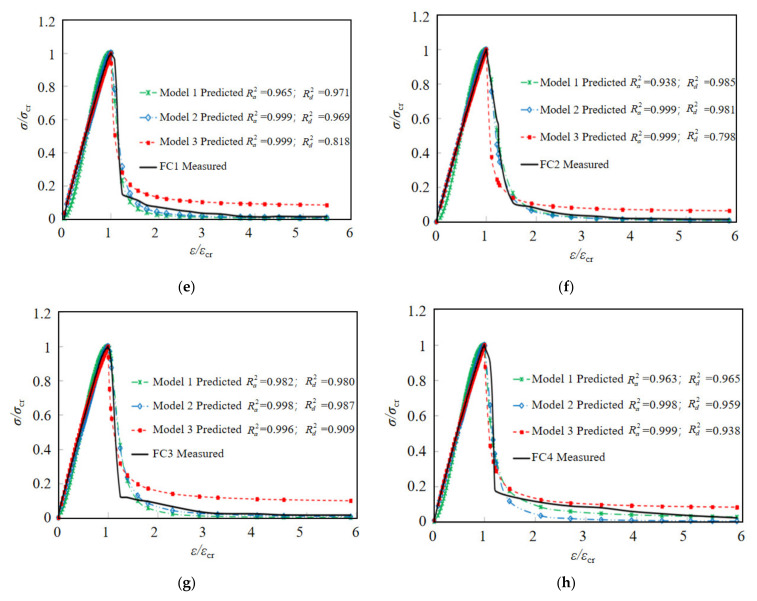
The fitting effect of stress–strain curves using different models. (**a**) C25, (**b**) C30, (**c**) C35, (**d**) C40, (**e**) FC1, (**f**) FC2, (**g**) FC3, (**h**) FC4.

In this paper, according to the curve characteristics of coral concrete, three models with different mathematical presentations are selected to calculate the ascending and declining stages of the curve, as shown in Table 9, and the accuracy of different models is shown in Figure 12 ($R_{a}^{2}$ and $R_{d}^{2}$) represent the fitting degree of the model in the ascending stages and descending stages, respectively).

As can be seen from Figure 12, in the ascending stage, the predicted results of model 2 and model 3 are in good agreement with the experimental data ($R_{a}^{2}\geq 0.99$), while in the descending stage, the predicted results of model 1 and model 2 are in good agreement with the experimental data ($R_{d}^{2}\geq 0.95$). Considering the coincidence with experimental curve and characteristic points of stress–strain curve, generally model 2 presents better results. Thus, the constitutive relationship of plain and PVA fiber-reinforced coral concrete can be recommend by the piecewise function model as the following equations, of which Equation (11) proposed by Yang [44] can be used to describe the ascending stage, and Equation (12) suggested by GB50010-2010 [47] can be used to describe the declining stage.

(11) $$y=\frac{(a+1)x}{a+x^{a+1}}$$

(12) $$y=\frac{x}{b{\left(x-1\right)}^{2}+x}$$

#### 3.2.4. Determination of Parameters and Model Validation

As important parameters of concrete, elastic modulus and peak strain are both critical parameters to reflect uniaxial compression behavior. Several studies have identified that there are many factors affecting the elastic modulus and peak strain of concrete, such as concrete strength and aggregate type [44,48].

**Figure 13:**
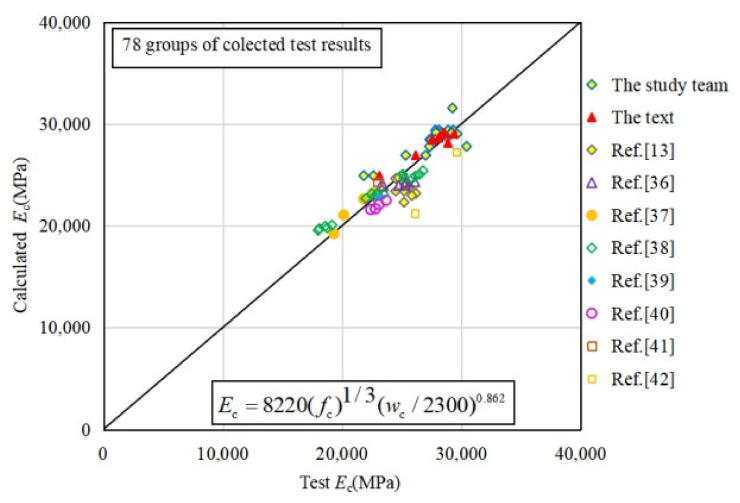
Comparison of test values and predicted values of elastic modulus.

**Figure 14:**
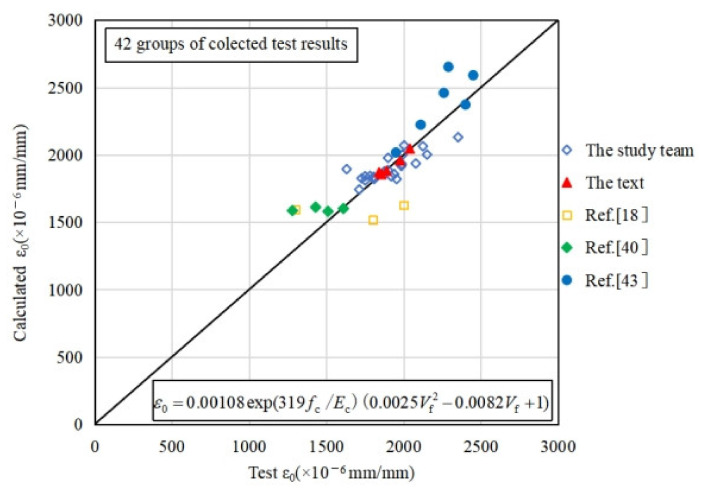
Comparison of test values and predicted values of peak strain.

As shown in Table 8 and discussed in Section 3.1.2 of this paper, the fiber has rarely influence on the elastic modulus of concrete. As shown in Figure 8a, the elastic modulus and the peak strain of coral concrete increases with an increase of concrete strength. Previous research has reported that the type of aggregate will also affect the elastic modulus of concrete, which is reflected by concrete density *w*_c_ [44]. Thus, based on the test data of plain and fiber-reinforced coral concrete from this research and previous literatures, the prediction approach of coral concrete elastic modulus *E*_c_ can be established with the prism compression strength *f*_c_ and concrete density *w*_c_ by regression analysis with 78 specimen test data, as shown in Equation (13). The validation of the prediction approach for elastic modulus is shown in Figure 13, with the determination coefficient *R*^2^ = 0.799. As shown in Figure 8a,c, the peak strain of coral concrete increases to some extent with increasing prism compression strength, and also increases obviously with increasing PVA fiber content. By regression analysis with 42 specimen test data, the prediction approach of peak strain for coral concrete can be established with the prism compression strength *f*_c_, elastic modulus *E*_c_ and fiber content *m*_f_. The validation of the prediction approach for peak strain is shown in Figure 14, with the determination coefficient *R*^2^ = 0.661. Among the experimental specimens, *f*_c_ representing the prism compression strength varies from 25 MPa to 75 MPa, *w*_c_ representing the concrete density varies from 1900 kg/m^3^ to 2300 kg/m^3^, and *m*_f_ representing the fiber content varies from 1 kg/m^3^ to 6.2 kg/m^3^.

(13) $$E_{c}=8220{\left(f_{c}^{}\right)}^{1/3}{\left(w_{c}/2300\right)}^{0.862}\;\;\;(R^{2}=0.799)$$

(14) $$\varepsilon_{cr}=0.00108\exp(319f_{c}^{}/E_{c})(0{.0025m}_{f}^{2}\;-\;0{.0082m}_{f}+1)\;\;\;(R^{2}=0.661)$$

As discussed above, Equations (11) and (12) are used to describe the uniaxial compression stress–strain behavior of plain and PVA fiber-reinforced coral concrete. Herein, the critical undetermined parameters *a* and *b* in Equations (11) and (12) needs to be determined for plain and PVA fiber reinforced coral concrete.

**Figure 15:**
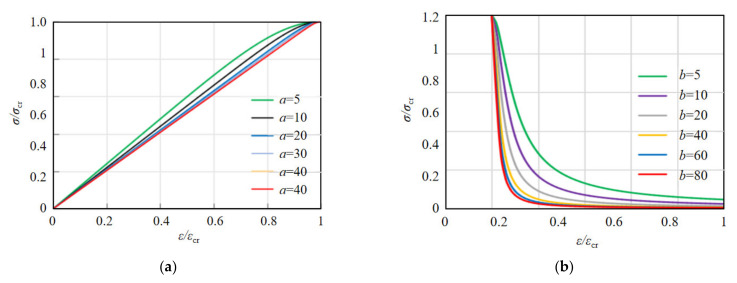
Effect of parameters “*a*” and “*b*” on *σ/σ*_cr_–*ε/ε*_cr_ curve. (**a**) Ascending stage, (**b**) Descent stage.

As can be seen from Figure 15, the smaller the value of parameter *a*, the plumper the curve, and the lower the strain growth rate of concrete in the loading process, then the more energy it would absorb. Similarly, the smaller the value of parameter *b*, the plumper the curve, the lower the slope of the curve in the unloading process, the slower the failure process, and the more energy released, which means the ductility of the coral concrete will be better. According to Figure 8 and Figure 15, it can be concluded that *a* and *b* should decrease with increasing coral concrete strength and increase with increasing fiber content.

**Figure 16:**
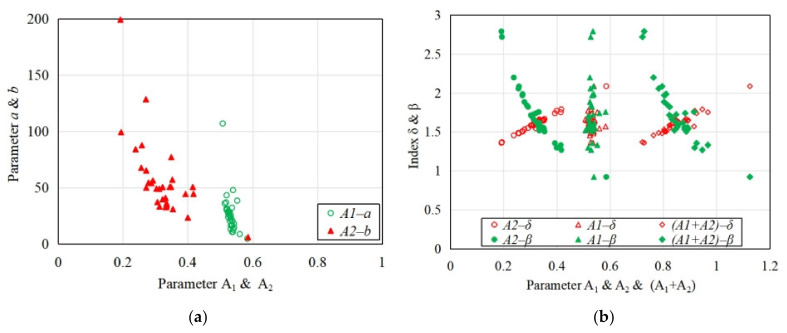
The relationship between model parameters and the toughness index of coral concrete. (**a**) The relationship between *a* and *A*_1_ (or *b* and *A*_2_), (**b**) The relationship between *δ*, *β* and *A*_1_, *A*_2,_ “*A*_1_ + *A*_2_”.

As shown in Figure 16, the test data of the specimens of coral concrete are collected and calculated to obtain the relationship between model parameters *a* or *b* and the toughness index of coral concrete. The results show that with the decrease of parameter *a* or *b*, the toughness parameters *A*_1_ or *A*_2_ tends to increase, simultaneously, *A*_2_ or “*A*_1_ + *A*_2_” is positively correlated with the toughness index *δ*, and negatively correlated with the brittleness index *β*, but the relationship between *A*_1_ and *δ* or *β* is not obvious. That is to say, with smaller parameters *a* and *b*, the corresponding *A*_1_ and *A*_2_ will become larger, and “*A*_1_ + *A*_2_” will also become larger, resulting in a larger toughness index *δ* and lower brittleness index *β*, indicating better deformation performance of the coral concrete. This is consistent with the conclusion of Figure 15. The results above from Figure 15 and Figure 16 show that the model parameters are related to the shape of the stress–strain curve.

**Figure 17:**
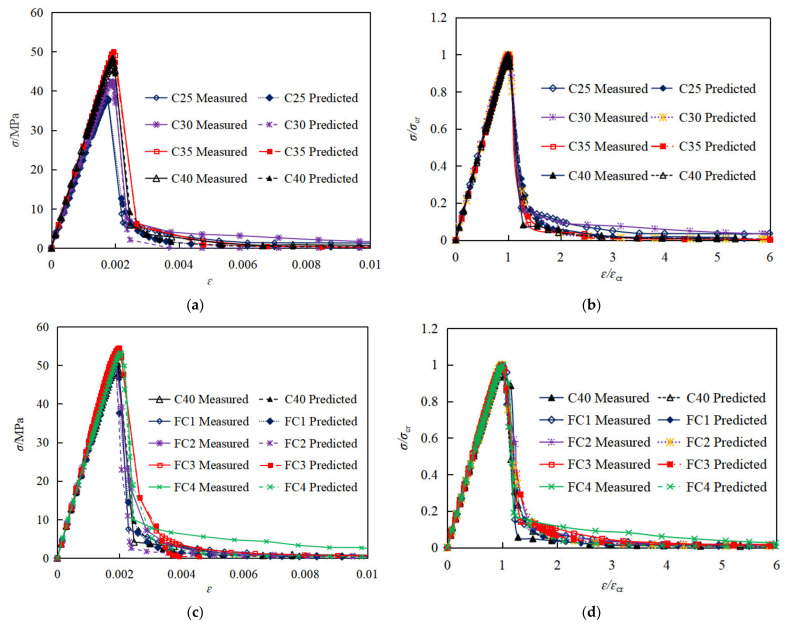
Fitting effect of model prediction. (**a**) Different strengths, (**b**) Different strengths (dimensionless), (**c**) Different fiber content, (**d**) Different fiber content (dimensionless).

Based on the analysis of the calculated results of the proposed model and the experimental data, the parameters *a* and *b* are both related to the fiber content and the compressive strength of coral concrete. In addition, the parameter *a* influencing the curve shape of the ascending stage may be also related to the elastic modulus *E*_c_ and peak strain *ε*_cr_ of coral concrete because they also influenced the curve shape, the toughness index *δ* and the brittleness index *β*. Therefore, the critical undetermined parameters *a* and *b* in Equations (11) and (12) are fitted by regression analysis of the experimental results, with consideration of potential influencing parameters including concrete strength *f*_c_, PVA fiber content *m*_f_, elastic modulus *E*_c_ and peak strain *ε*_cr_. The stress–strain expression calculated by the proposed model for all the specimen groups tested in this work and the determination approach for critical parameter *a* and *b* are shown in Table 10. In addition, the predicted stress–strain curves for experimental specimens using Equations (11) and (12) and the proposed the determination approach for critical parameter *a* and *b* in Table 10 is shown in Figure 17.

As shown in Table 10 and Figure 17, the coefficients of determination ($R^{2}$) of both the ascending and descending stages are greater than 0.95, and the predicted results of the proposed model are in good agreement with the experimental results. Thus, it can be concluded that the proposed model and the suggested determination approach of parameter *a* and *b* are capable of accurately describing the constitutive relationship of plain and PVA fiber-reinforced coral concrete.

**Figure 18:**
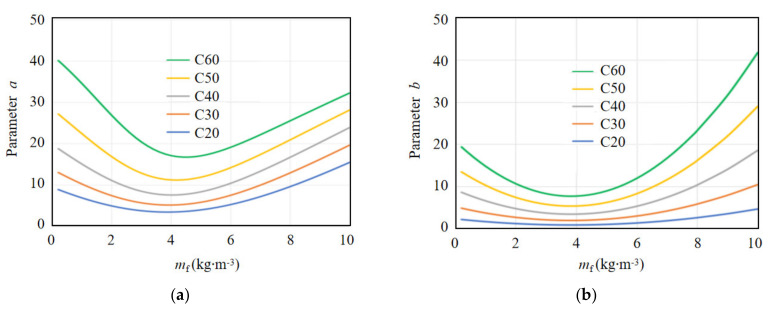
The relationship between model parameters and fiber content and concrete strength. (**a**) Parameter a, (**b**) Parameter b.

In addition, the best PVA fiber content for coral concrete with different compressive strength is also analyzed using Equations (11) and (12) and the suggested determination approach of parameter *a* and *b* in Table 10, as shown in Figure 18. With an increase of the concrete strength, the parameters *a* and *b* increase correspondingly, meaning that the brittleness index *β* increases and the toughness index *δ* decreases. As is known from experimental results, with increasing concrete strength, the elastic modulus and the brittleness of concrete also increases, evidencing that the prediction approach gives good consideration of the influence of concrete strength. In addition, with the increase of PVA fiber content, the parameter *a* and *b* decrease firstly when $V_{f}$ does not exceed 4 kg/m^3^, and then increase after *m*_f_ exceeds 4 kg/m^3^. As presented above, the smaller parameter *a* and *b*, the better toughness and smaller brittleness of coral concrete. That is to say, the best content of PVA fiber may be about 4 kg/m^3^, which coincides with the experimental results. This phenomenon could be attributed to the bridging effect of PVA fiber, which effectively prevents the expansion of transverse cracks and consumes energy when fracture. However, when the fiber content is too large, it would cause fiber winding and clusters, forming weak areas in the coral concrete, resulting in the reduction of fiber toughening effect. As shown in Figure 18, with an increase of concrete strength, the best content of PVA fiber increases slightly around 4 kg/m^3^. Therefore, the best content of PVA fiber can be suggested as 4 kg/m^3^ regardless of concrete strength.

## 4. Conclusions

1. According to a certain proportion of silica fume, slag, P.O. 42.5 cement, coral coarse and fine aggregate, artificial sea water, etc., coral aggregate–seawater concrete with the compressive strength of 30 to 50 MPa and good performance can be prepared.
2. With an increase of PVA fiber content, the slump and dispersion of coral concrete decreases significantly. In order to ensure the working performance of coral concrete, it is suggested that the PVA fiber content of coral concrete should not exceed 5 kg/m^3^ in marine environment construction, and not exceed 2 kg/m^3^ if using pumping construction.
3. With increasing strength of coral concrete, the elastic modulus, Poisson’s ratio and peak strain increase, but the residual stress and the toughness decrease.
4. PVA fiber effectively improves the compression strength of coral concrete, as well as the ductility and mechanical properties after peak load. Compared with the reference coral concrete, the maximum growth rate of cubic compressive strength *f*_cu_ is up to 9.9%. The optimal amount of PVA fibers to optimize mechanical properties of coral concrete is about 2–3 kg/m^3^.
5. PVA fiber has remarkable effect on improving the toughness of coral concrete. When the fiber content ranges from 0.8 to 6.5 kg/m^3^, the toughness index *δ* of coral concrete increases by 2.9% to 10.1%, and the brittleness index *β* decreases by 15.3–28.8%.
6. The constitutive relationship of plain and PVA fiber-reinforced coral concrete can be described by the recommended piecewise function model and the proposed approach to determining critical parameter *a* and *b*. In addition, through analysis of the model, the optimal PVA fiber content of coral concrete may be 4 kg/m^3^.
7. To meet the requirements of structure design and environmental benefits, it plays a very important guiding role in the engineering application of coral concrete to optimize the mix ratio design and improve the corrosion resistance, high temperature resistance and drying shrinkage resistance of coral concrete structure. However, more validation experiments are needed though.

## Figures and Tables

**Table 1:** Physical property of coral aggregate.

Type	Bulk Density (kg/m^3^)	Apparent Density (kg/m^3^)	Water Absorption (%)	Cylindrical Strength (MPa)	Void Content (%)
Coarse aggregate	888	1870	12	3.8	52.5
Fine aggregate	1392	2380	10	-	42.5

**Table 2:** Chemical composition of cementitious materials.

Test Project	W%							Specific Surface Area (m^2^·kg^−1^)	Loss of Ignition	Density (kg/m^3^)
	SiO_2_	Al_2_O_3_	Fe_2_O_3_	CaO	MgO	SO_3_	LOI			
OPC	22.47	4.83	2.97	59.28	1.97	2.39	-	-	2.75	3100
SG	33.65	15.3	0.46	35.42	10.2	0.70	0.15	424	0.71	2940
SF	95.70	0.54	0.06	0.76	0.54	0.01	1.32	18,465	2.95	330

**Table 3:** Physical properties of PVA fiber.

Length (mm)	Diameter (μm)	Density (kg/m^3^)	Young Modulus (Gpa)	Tensile Strength (Mpa)	Elongation (%)
11–13	42–46	1290	33.8	1430	6–11

**Table 4:** Chemical composition of artificial seawater (g/L).

NaCl	MgCl_2_·6H20	Na_2_SO_4_	CaCl_2_	KCl	NaHCO_3_
22.2	11.3	3.86	1.08	0.75	0.21

**Table 5:** Coral concrete mix proportions.

Groups	Mix Proportions (kg/m^3^)								Total *w/b*
	OPC	S95	SF95	Coarse Aggregate	Coral Sand	Total Water	SP	Fiber	
C25	255	82	27	729	729	246	2.9	0	0.68
C30	288	93	31	709	709	241	3.7	0	0.58
C35	420	135	45	700	700	235	7.2	0	0.39
C40	480	90	30	625	625	220	10	0	0.37
FC1	480	90	30	625	625	220	10	0.8125	0.37
FC2	480	90	30	625	625	220	10	1.625	0.37
FC3	480	90	30	625	625	220	10	3.25	0.37
FC4	480	90	30	625	625	220	10	6.5	0.37

Note: “*w/b*” is water-binder ratio, where, “*w*” denotes dosage of water in the mix, in kg/m^3^; *b* = m_C_ + m_SG_ + m_SF_, m_C_, m_SG_ and m_SF_ represent the dosage of OPC, SG, SF, respectively, in kg/m^3^.

**Table 6:** Classification of workability (mm).

Slump	10–40	50–90	100–150	160–210	≥220	---
Grades	S1	S2	S3	S4	S5	---
Dispersion	≤340	350–410	420–480	490–550	560–620	≥630
Grades	F1	F2	F3	F4	F5	F6

Note: Slump and dispersion are quantitative indexes to measure the workability of concrete, and the greater the slump and dispersion, the better the workability. From S1 to S5 or from F1 to F6, the workability of concrete is getting better.

**Table 7:** Workability of coral concrete.

Groups	C25	C30	C35	C40	FC1	FC2	FC3	FC4
Slump T_i_/mm	190	220	250	260	250	230	160	65
Dispersion K_i_/mm	430	490	610	700	690	565	300	200
Slump grade	S4	S5	S5	S5	S5	S5	S4	S2
Dispersion grade	F3	F4	F5	F6	F6	F5	F1	F1

Note: using the test method in Figure 3, the slump value $T_{i}\leq 300\mathrm{mm}$ and the dispersion value $K_{i}\geq 200\mathrm{mm}$.

**Table 8:** Measured mechanical properties of coral concrete.

Groups	$m_{f}{(\mathrm{kg}/m}^{3})$	$f_{\mathrm{cu},100,28d}\left(\mathrm{MPa}\right)$	$\sigma_{\mathrm{cr}}(\mathrm{MPa})$	$\varepsilon_{c}(\mu \varepsilon )$	$E_{c}(\mathrm{MPa})$	μ	$\sigma_{r}\left(\mathrm{MPa}\right)$	$w_{c}{(\mathrm{kg}/m}^{3})$
C25	0	30.5	38.1	1754	24,960	0.229	8.7	2040
C30	0	35.5	42.4	1874	26,179	0.224	7.6	2140
C35	0	41.8	50.0	1940	28,886	0.242	4.8	2110
C40	0	45.9	48.3	1889	28,542	0.242	3.9	2220
FC1	0.8125	48.1	46.8	1856	28,093	0.237	7.5	2214
FC2	1.625	51.2	49.8	1841	29,406	0.302	7.1	2193
FC3	3.25	54.9	51.8	1977	28,488	0.286	6.8	2171
FC4	6.5	50.3	49.5	2040	27,548	0.252	9.1	2150

Note: $m_{f}$ is fiber content; $f_{\mathrm{cu},100,28d}$ is the cube compressive strength of concrete at 28 days with the size of 100 mm × 100 mm × 100 mm; $\sigma_{\mathrm{cr}}$ is the peak stress of concrete with the size of 100 mm × 100 mm × 300 mm; $\varepsilon_{c}$ is peak strain; $E_{c}$ is elastic modulus; *μ* is Poisson’s ratio; $\sigma_{r}$ is residual stress; $w_{c}$ is apparent density.

**Table 9:** Different models of stress–strain curve equation.

Section	Numbers	Mathematical Model	Proposer/Literature
Ascending stage	model 1	$y=ax+(3-2a)x^{2}+(a-2)x^{3}$	Guo [43]
	model 2	$y=\frac{(a+1)x}{\left(a+x^{a+1}\right)}$	Yang [44]
	model 3	$y=xe^{a(1-x)}$	Sahlin [45] modified
Declining stage	model 1	$y=\frac{x}{b{\left(x-1\right)}^{c}+x}$	Zhao [46]
	model 2	$y=\frac{x}{b{\left(x-1\right)}^{2}+x}$	GB50010-2010 [47]
	model 3	$y=\frac{x}{b{\left(x-1\right)}^{}+x}$	Da [27]

Notes: *x* = *ε*_c_/*ε*_cr_, *y* = *σ*_c_/*σ*_cr_, *σ*_cr_, *ε*_cr_ is the peak stress and peak strain; *a*, *b* and *c* are the undetermined parameters of the model.

**Table 10:** Determination of parameters *a* and *b*.

Types	Model	Parameter	C25	C30	C35	C40	FC1	FC2	FC3	FC4	Key Parameter Value
Ascend stage	$y=\frac{\left(a+1\right)}{a+x^{a+1}}$	*a*	21.84	22.48	30.64	28.53	21.62	17.52	14.36	18.47	$\begin{array}{l} a=\frac{\left(0.1642m_{f}^{2}-1.213m_{f}+3.2\right)}{\left(1-f_{c}/(E_{c}\varepsilon_{\mathrm{cr}})\right)}\\ R^{2}=0.9407 \end{array}$
		*R* ^2^	0.999	0.999	0.999	0.999	0.999	0.999	0.998	0.998	
Decline stage	$y=\frac{x}{b{\left(x-1\right)}^{2}+x}$	*b*	39.68	50.67	65.80	51.64	48.75	33.29	29.50	45.41	$\begin{array}{l} b=(0.0011m_{f}^{2}-0.0083m_{f}+0.0256)f_{c}^{2}\\ R^{2}=0.7676 \end{array}$
		*R* ^2^	0.990	0.989	0.985	0.968	0.969	0.981	0.987	0.959	

Note: *R*^2^ refers to the fitting degree of regression line to corresponding data.

## Data Availability

All data reported in this paper is contained within the manuscript.
